# Visualization and prediction of the pleura and thoracic duct: elucidation of changes due to respiration using arterial landmarks and CT images

**DOI:** 10.1007/s00276-025-03582-3

**Published:** 2025-02-07

**Authors:** Niina Hirano, Satoru Muro, Junichi Tsuchiya, Keiichi Akita

**Affiliations:** 1 Department of Clinical Anatomy, Institute of Science Tokyo, Bunkyo, Tokyo Japan; 2 Department of Diagnostic Radiology and Nuclear Medicine, Institute of Science Tokyo, Bunkyo, Tokyo Japan

**Keywords:** Thoracic duct, Pleura, Anatomy, Computed tomography image, Respiration

## Abstract

**Purpose:**

The thoracic duct uses the pulsation of accompanying arteries to facilitate lymphatic flow. However, the lymphatic flow mechanism cannot be explained when it does not accompany the arteries. This study aimed to clarify the anatomical position of the thoracic duct and surrounding structures and determine the differences in the thoracic duct length and course during inspiration and expiration.

**Methods:**

Six cadavers were dissected to observe the positional relationship between the thoracic duct and surrounding structures. Image sequences of anatomical sections from the Visible Korean Human Open Resource were observed and reconstructed to understand their three-dimensional positioning. Inspiratory and expiratory computed tomography scans were used to measure and examine respiratory variations in the distance between the arterial landmarks to predict the thoracic duct length.

**Results:**

The thoracic duct accompanied the arteries for most of its course and was sandwiched between the arteries and pleura, entering the mediastinum. However, there was an area on the cranial side of the aortic arch where the thoracic duct did not accompany the arteries. The distance between the arterial landmarks in this area, which approximate the thoracic duct length, was significantly longer during inspiration (39.3 ± 7.81 mm) than during expiration (31.49 ± 7.01 mm).

**Conclusion:**

This study suggests that the pleura entering the mediastinum pushes the thoracic duct toward the arteries to promote lymphatic flow generation by arterial pulsation. Additionally, this study suggests that the lymphatic flow in the thoracic duct is generated by the expansion and contraction of the thoracic duct with respiratory movement.

## Introduction

The thoracic duct is described as the largest lymphatic vessel in the human body, running from the cisterna chyli at the L2 level to the neck [12, 19]. It passes through the aortic hiatus of the diaphragm and enters the posterior mediastinum. In the mediastinum, the thoracic duct ascends between the descending aorta and azygous vein and moves leftward posterior to the esophagus around the T5 level. The thoracic duct moves anteriorly along the aortic arch, ascends posterior to the left subclavian artery, and curves laterally to open into the vein (the left subclavian vein, left internal jugular vein, or left venous angle). Studies using macroscopic anatomy and medical imaging have reported that the thoracic duct largely accompanies arteries such as the aorta, common carotid artery, and subclavian artery [2, 9, 13, 14]. Lymph flow within the valvular vessels is facilitated by compression from the accompanying arteries [8, 16], and the thoracic duct accompanies the great arteries, suggesting that lymphatic flow within the thoracic duct is also generated by the arteries. The only exception is the region cranial to the aortic arch, where the thoracic duct does not accompany the arteries, indicating that the lymphatic flow in this region cannot be explained by arterial compression alone.

Although little is known about the positional relationships between the thoracic duct and structures other than the arteries, the parietal pleurae have been reported to partially enter the mediastinum and separate the thoracic duct from the esophagus [17]. Therefore, we hypothesized that the thoracic duct has a structural relationship not only with the arteries, but also with the pleurae. In addition, the diameter of the thoracic duct opening changes with respiration [5], and we considered that morphological changes of the thoracic duct caused by respiration may affect the lymphatic flow.

This study aimed to clarify the detailed positional relationship between the thoracic duct and surrounding structures, especially the pleura, and determine the differences in the course and length of the thoracic duct during inspiration and expiration. This study may provide insights into the relationship between the physiological function of the thoracic duct and respiration.

## Materials and methods

### Macroscopic examination

Six Japanese cadavers (two males and four females; mean age at death and standard deviation, 78.8 ± 15.5 years) donated to the Department of Clinical Anatomy were used for the macroscopic anatomy. The format of the donation documents was consistent with the guidelines of the Act on Body Donation for Medical and Dental Education Law of Japan. Donors declared their willingness to donate their remains for educational purposes before passing away. This voluntary cadaveric donation system is applied throughout Japan, and our study fully complies with the Japanese laws entitled “The Act on Body Donation for Medical and Dental Education” (Act No. 56 of 1983). All the cadaveric specimens were fixed in 8% formalin and preserved in 30% ethanol.

Macroscopic examination was performed to reveal the course of the thoracic duct and its positional relationship with the surrounding structures, particularly the arteries and pleurae. The mediastinal region was obtained from the cadavers by removing the head, upper limbs, including the scapulae, and caudal side of the diaphragm using a diamond band pathology saw (EXAKT 312; EXAKT Advanced Technologies GmbH, Norderstedt, Germany). The skin on the ventral side, subcutaneous soft tissue, and muscles around the chest wall were excised. To observe the structures in the mediastinum, the anterior side of the chest wall and bilateral clavicles were excised, and the bilateral lungs and heart were identified. The lungs were then excised, leaving a small portion of the hilum to expose the entire heart covered by the pericardium. The positional relationships between the thoracic duct and surrounding structures (esophagus, vessels, and pleura) were observed during dissection.

### Observation of anatomical sections

Image sequences of anatomical sections from Visible Korean Human (VKH) Open Resource (33-year-old male) (http://anatomy.co.kr/) [4, 11, 21] were used to clarify the position of structures at different heights. To observe the axial plane, 276 cross-sectional images at 1 mm intervals at the C7 to T12 level were used. The thoracic duct and surrounding major structures such as the arteries, veins, esophagus, trachea/ main bronchi, pleurae, and vertebrae, were identified. The change in the thoracic duct position and its positional relationships to surrounding structures were observed.

### Three-dimensional reconstruction

Three-dimensional reconstruction was conducted to understand the course of the thoracic duct and observe its positional relationship with the surrounding structures in all directions. Major structures identified during the observation of the anatomical sections were manually segmented from 276 cross-sectional images, and three-dimensional reconstruction was performed using Srf II software (ver. R. 11.00.00.0-H, Ratoc System Engineering, Tokyo, Japan; http://www.ratoc.com/eng/index.html).

### Measurement of respiratory variations in the distance between the arterial landmarks on computed tomography (CT) images

**Fig. 1:**
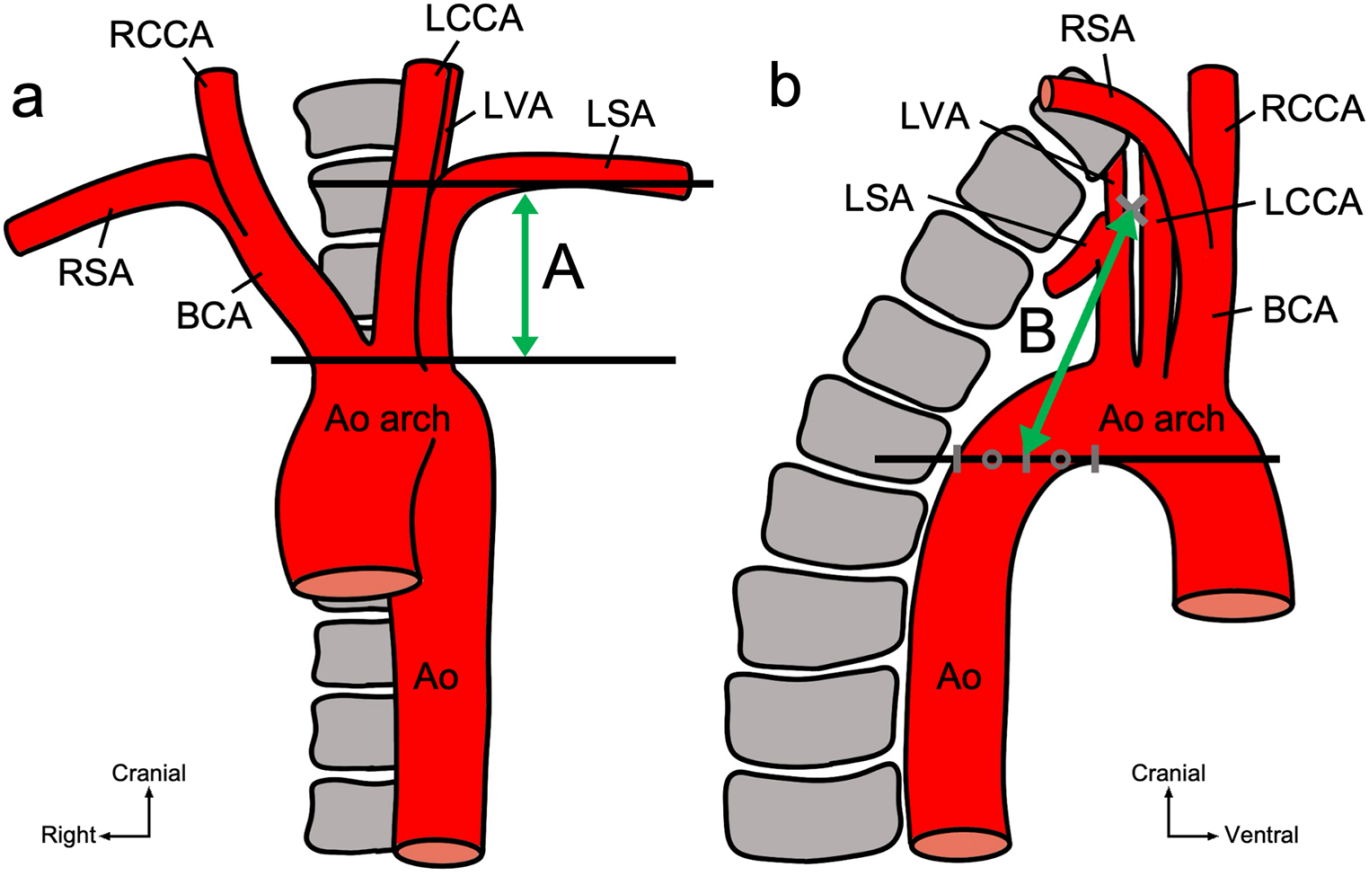
Schematic representation of the thoracic duct measured on computed tomography images. **a** The length in the coronal plane (double arrow: A) is defined as the vertical distance between the superior edge of the aortic arch and the inferior edge of the horizontal portion of the left subclavian artery. **b** Length in the sagittal plane (double arrow: B) is defined as the distance between two points: the point where the left subclavian artery curves outward and the left vertebral artery branches off, and the midpoint of the inferior edge of the aortic arch and the posterior edge of the descending aorta on the horizontal line at the level of the inferior edge of the aortic arch. *Ao* aorta, *BCA* brachiocephalic artery, *LCCA* left common carotid artery, *LSA* left subclavian artery, *LVA* left vertebral artery, *RCCA* right common carotid artery, *RSA* right subclavian artery

**Fig. 2:**
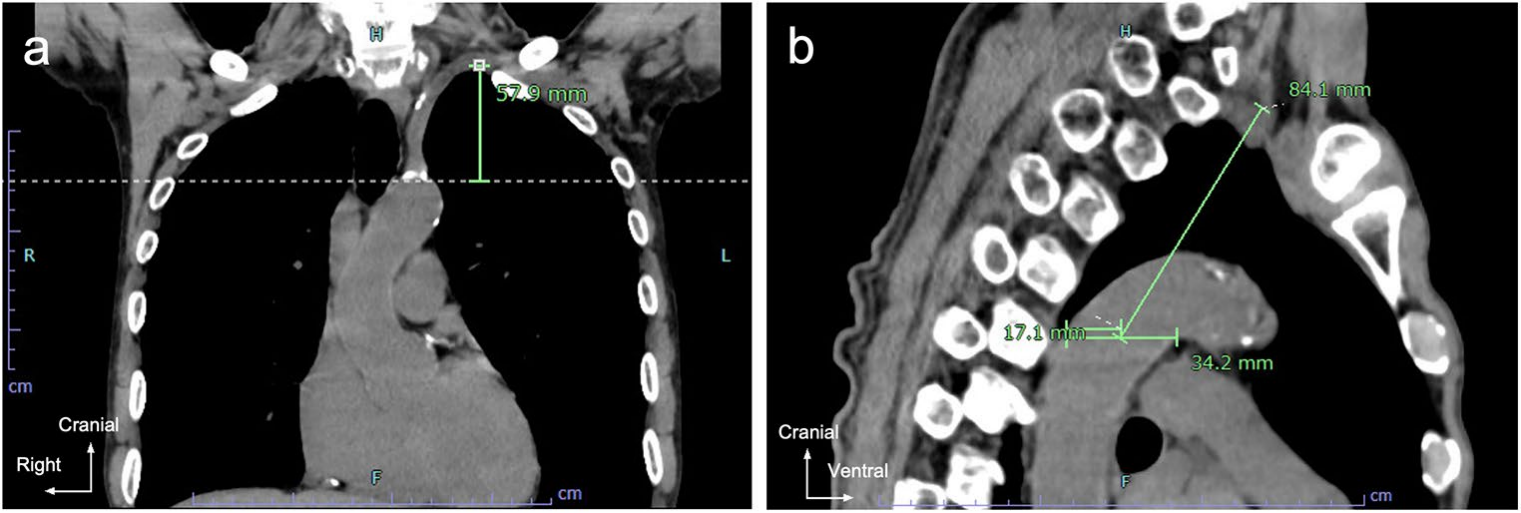
Chest computed tomography images during inspiration. The green line segments indicate the measured distance between the landmarks and correspond to the double arrows in Fig. 1. **a** Coronal plane. **b** Sagittal plane

The distance between two points approximating the thoracic duct length was measured on CT images to investigate the effect of respiratory movement on the thoracic duct length in the upper mediastinum. Fifty chest CT imaging examinations, including expiratory CT, performed at Tokyo Medical and Dental University Hospital between January 1, 2022 and September 30, 2023 were used. (Tokyo Medical and Dental University Hospital was renamed to Institute of Science Tokyo Hospital on October 1, 2024). The scan parameters for SOMATOM Force (Siemens Healthinees AG, Germany) were as follows: detector collimation 196; matrix size 512; field of view (FOV) adjusted according to the patient’s body size; pitch 1.2; gantry rotation time 0.5 s/rot; slice thickness 1 mm. The scan parameters for Canon Aquilion 64 were as follows: detector collimation 64; matrix size 512; field of view (FOV) adjusted according to the patient’s body size; pitch not specified; gantry rotation time not specified; slice thickness 1 mm. The scan parameters for SOMATOM Edge Plus (Siemens Healthinees AG, Germany) were as follows: detector collimation 64; matrix size 512; field of view (FOV) adjusted according to the patient’s body size; pitch 1.2; gantry rotation time 0.5 s/rot; slice thickness 1 mm. We have excluded cases with significant motion artefacts and those in which there was no change in absorption value between inspiratory and expiratory phases, resulting in an inability to obtain expiratory CT images. As it is difficult to identify the thoracic duct on plain CT images, landmarks based on the surrounding arteries were established for measurement. The landmarks were determined based on consistent points observed in the path of the thoracic duct during macroscopic examination. The distance between two points approximating the thoracic duct length was measured (SYNAPSE SAI viewer, Fujifilm, Tokyo, Japan) in the coronal (Figs. 1a and 2a) and sagittal (Figs. 1b and 2b) planes during inspiration and expiration. The vertical distance between the superior edge of the aortic arch and the inferior edge of the horizontal portion of the left subclavian artery was measured in the coronal plane (Fig. 1a, A). In the sagittal plane, the distance was measured from the point where the left subclavian artery curves outwards and the left vertebral artery branches off to the midpoint of the inferior edge of the aortic arch and the posterior edge of the descending aorta on a horizontal line at the level of the inferior edge of the aortic arch (Fig. 1b, B).

### Statistical analysis

Means and standard deviations were calculated for inspiratory changes, expiratory changes, and inspiratory and expiratory differences in the coronal and sagittal planes. R software for Windows version 4.3.2 (The R Foundation for Statistical Computing, Vienna, Austria) was used for the t-test to confirm significant inspiratory and expiratory differences in the coronal and sagittal planes.

## Results

### Macroscopic examination

**Fig. 3:**
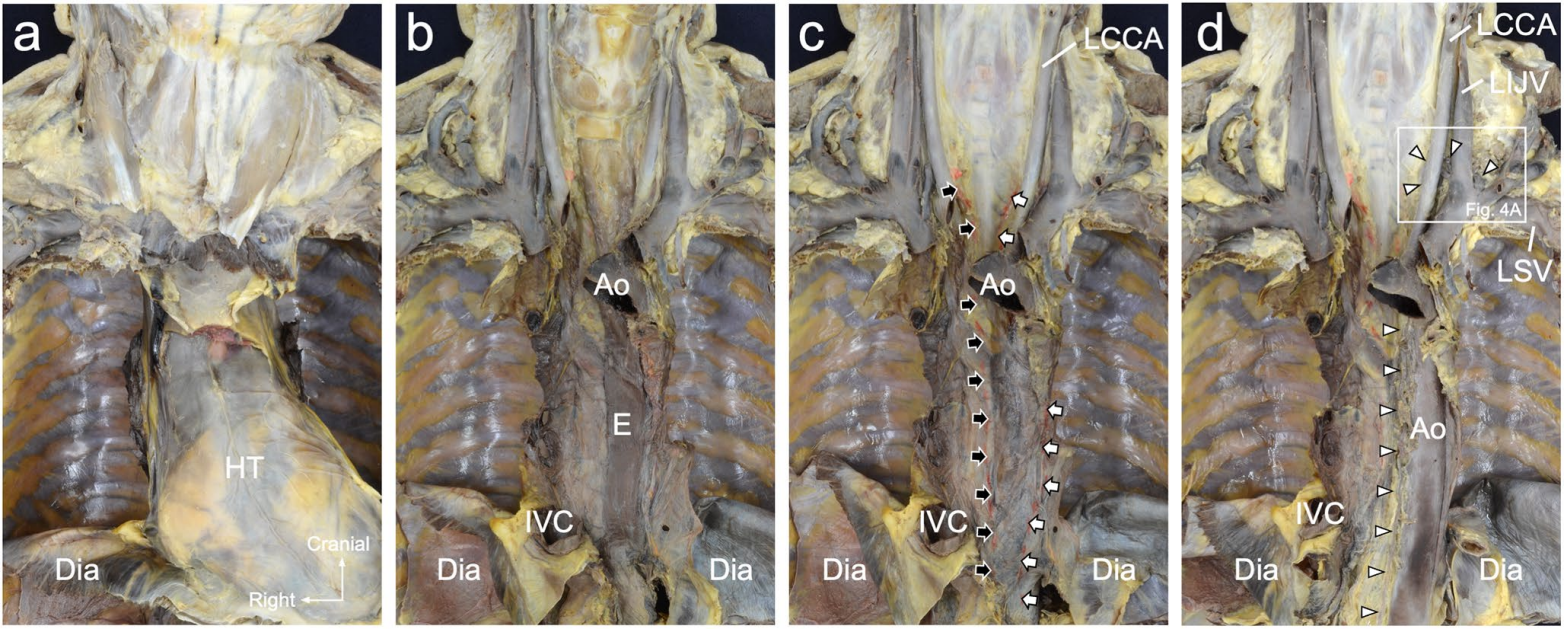
Macroscopic observation of the chest from the ventral aspect. **a** The skin, muscle, ventral side of the chest wall, and lungs are excised. **b** The heart is excised to visualize the esophagus. **c** The esophagus is excised to visualize the aorta. The bilateral pleurae are inserted into the mediastinum (black arrows: medial margin of the right pleura; white arrows: medial margin of the left pleura). **d** The membranous structure of the connective tissue on the ventral side of the aorta is excised. The thoracic duct (white arrowheads) ascends along the right side of the aorta with branches and anastomoses. *Ao* aorta, *Dia* diaphragm, *E* esophagus, *HT* heart, *IVC* inferior vena cava, *LCCA* left common carotid artery, *LIJV* left internal jugular vein, *LSV* left subclavian vein

After identifying the heart in the mediastinum (Fig. 3a), the heart and trachea/bronchus were excised, and the esophagus was observed to run longitudinally in the mediastinum behind the heart (Fig. 3b). After excising the esophagus, the descending aorta was observed (Fig. 3c). The bilateral pleurae were entering the mediastinum, posterior to the esophagus and anterior to the aorta. In the lower mediastinum, the bilateral pleurae were strongly inserted to cover the descending aorta. By contrast, superior to the aortic arch, the left pleura extended medially posterior to the left subclavian artery, branching from the aorta. After excising the thin membranous structure covering the anterior surface of the descending aorta, the thoracic duct running on the right side of the descending aorta was observed (Fig. 3d). Superior to the aortic arch, the thoracic duct ran medial to the left common carotid artery and terminated at the left venous angle (between the left internal jugular vein and left subclavian vein).

**Fig. 4:**
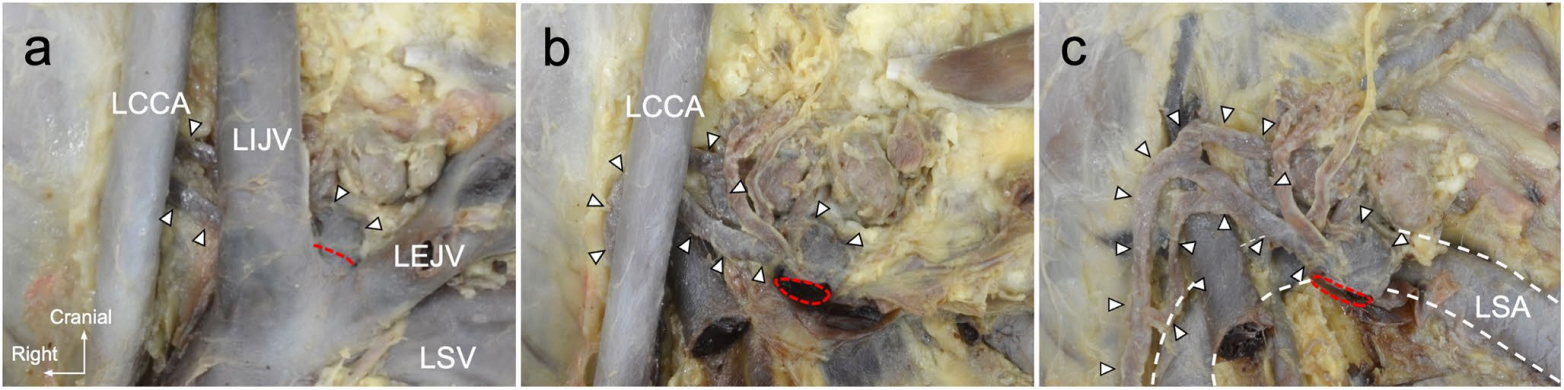
Dissection of the thoracic duct and cervical vessels. A magnified image of the box region in Fig. 3d. **a** The thoracic duct opens into the left venous angle (between the left internal and external jugular veins). **b** The vein constructing the left venous angle is excised to show the positional relationship between the thoracic duct and arteries. **c** The left common carotid artery is excised. The thoracic duct is located behind the left common carotid artery and above the left subclavian artery *LCCA* left common carotid artery, *LEJV* left external jugular vein, *LIJV* left internal jugular vein, *LSA* left subclavian artery, *LSV* left subclavian vein

Upon closer examination of the thoracic duct superior to the aortic arch, it ascended along the left common carotid artery (Fig. 4b), changed direction to the left with the left subclavian artery, and terminated at the left venous angle (Fig. 4c). In two of the six cadavers, the thoracic duct branched as it ran along the left common carotid and left subclavian arteries.

### Observation of anatomical sections

**Fig. 5:**
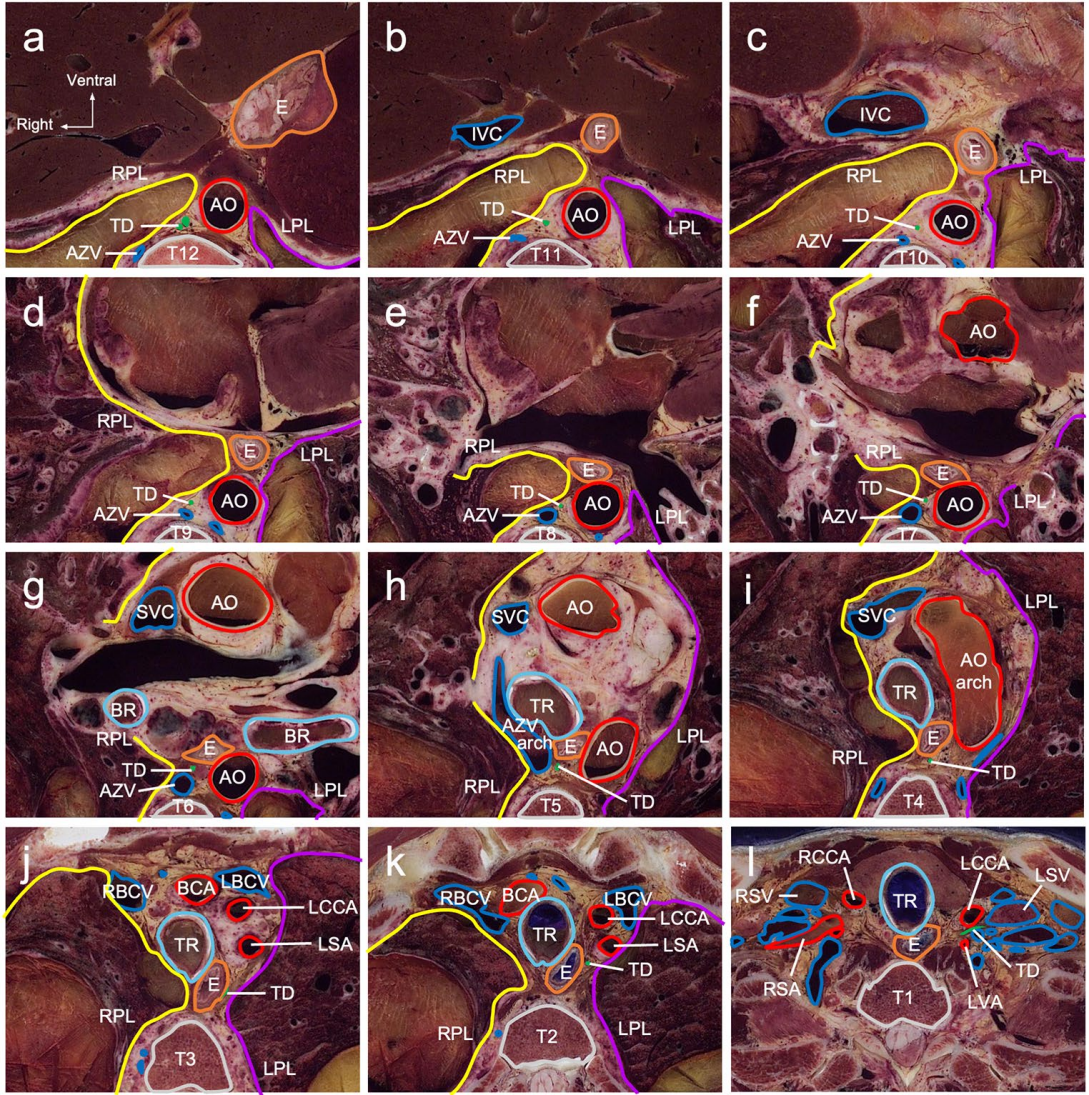
Cross-sectional image around the mediastinum on each vertebral level. **a-e** The thoracic duct is located on the right side of the aorta and dorsal side of the right pleura in the lower mediastinum. **f-h** The thoracic duct is surrounded by the esophagus, aorta, and azygos veins. **i**,** j** The thoracic duct shifts from the dorsal to the left ventral side of the esophagus, running between the esophagus and the left pleura. **k** The thoracic duct moves toward the left subclavian and left common carotid arteries. **l** The thoracic duct passes between the left common carotid artery and left vertebral artery and opens into the left venous angle *AO* aorta, *AZV* azygos vein, *BCA* brachiocephalic artery, *BR* bronchus, *E* esophagus, *IVC* inferior vena cava, *LBCV* left brachiocephalic vein, *LCCA* left common carotid artery, *LPL* left pleura, *LSA* left subclavian artery, *LSV* left subclavian vein, *LVA* left vertebral artery, *RBCV* right brachiocephalic vein, *RCCA* right common carotid artery, *RPL* right pleura, *RSA* right subclavian artery, *RSV* right subclavian vein, *SVC* superior vena cava, *TD* thoracic duct, *TR* trachea, *V* thoracic vertebra

Following the lymphatic flow of the thoracic duct, observations are described from caudal to cranial. At the level of the 12th and 11th thoracic vertebrae (T12 and T11), the thoracic duct was located posterior to the right pleura and accompanied by the aorta (Fig. 5a, b). From the level of T10 to T7, the thoracic duct ran between the aorta and azygous vein (Fig. 5c-f). At T6 level, the thoracic duct was surrounded by the aorta, azygous vein, and esophagus (Fig. 5g). The azygous vein and aortic arches were identified at the T5 and T4 levels, respectively (Fig. 5h, i). Thus, the azygous vein and aorta shifted anteriorly away from the thoracic duct. Superior to the T3 level, the thoracic duct moved anteriorly upward, trapped between the esophagus and left pleura (Fig. 5j), and moved toward the left subclavian artery at the T2 level (Fig. 5k). At the T1 level, the thoracic duct passed between the left common carotid artery and left vertebral artery (Fig. 5l), a branch of the left subclavian artery, and terminated at the left venous angle.

### Three-dimensional reconstruction

**Fig. 6:**
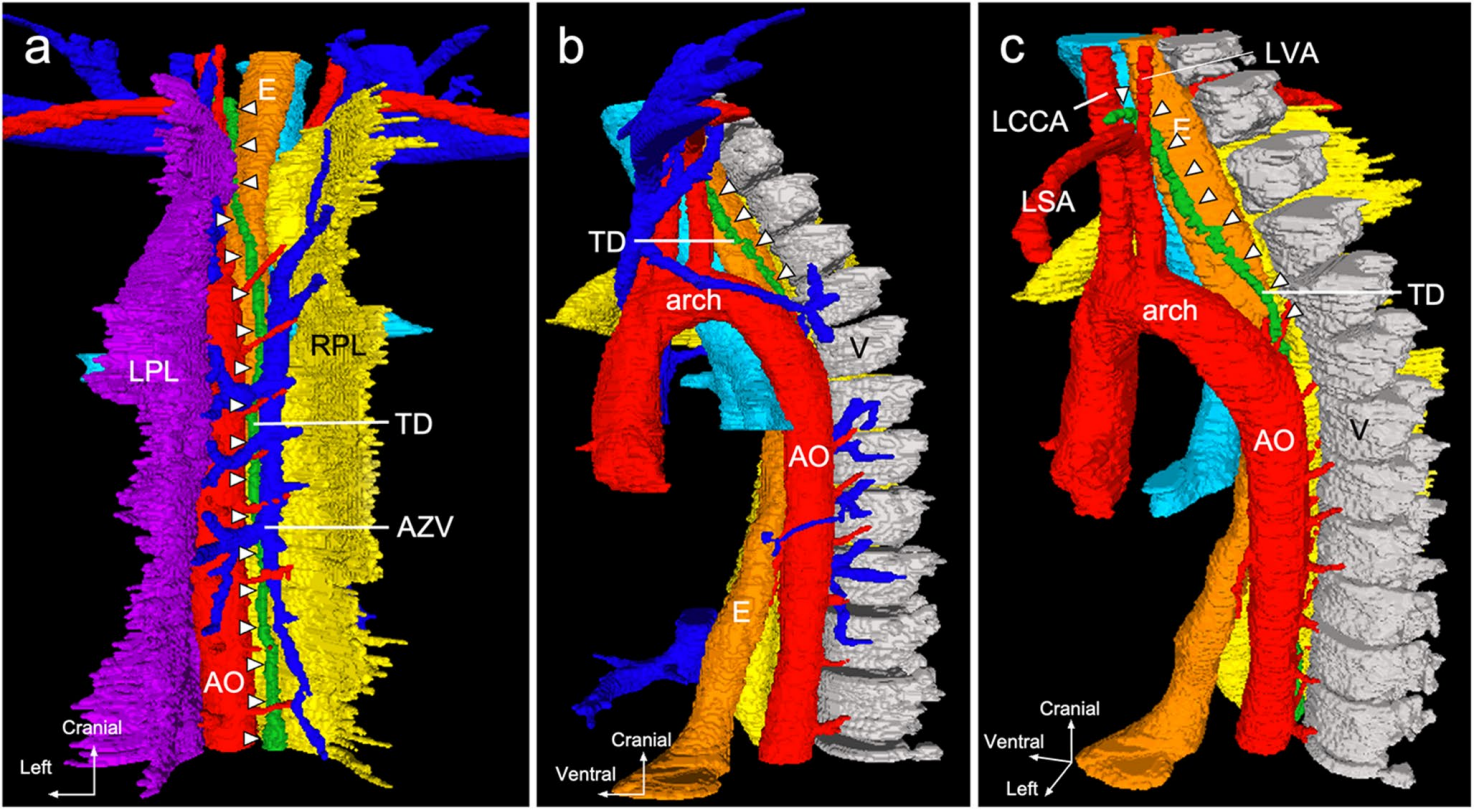
Three-dimensional reconstruction image. **a** The thoracic duct ascends to the right side of the aorta and dorsal side of the right pleura and is inserted into the mediastinum. **b** The thoracic duct ascends along the esophagus on the cranial side of the aortic arch. **c** In the cervical region, the thoracic duct passes between the left common carotid and vertebral arteries and opens into the left venous angle *AO* aorta, *AZV* azygos vein, *E* esophagus, *LCCA* left common carotid artery, *LPL* left pleura, *LSA* left subclavian artery, *LVA* left vertebral artery, *RPL* right pleura, *RSA* right subclavian artery, *TD* thoracic duct, *V* thoracic vertebra

Three-dimensional reconstruction was performed using cross-sectional images. In the lower mediastinum, the thoracic duct ran along the aorta, posterior to the right pleura entering the mediastinum (Fig. 6a). In other words, the pleura extended into the mediastinum to keep the thoracic duct and aorta close to each other. Superior to the aortic arch, the thoracic duct ran anterior to the left pleura and entered the mediastinum. Away from the aortic arch, the thoracic duct ascended along the esophagus, moving posteriorly to anteriorly (Fig. 6b). The anteriorly displaced thoracic duct passed between the left common carotid and subclavian arteries, deviated to the left lateral along the left subclavian artery (Fig. 6c), and terminated at the left venous angle.

### Thoracic duct independent of the arteries

**Fig. 7:**
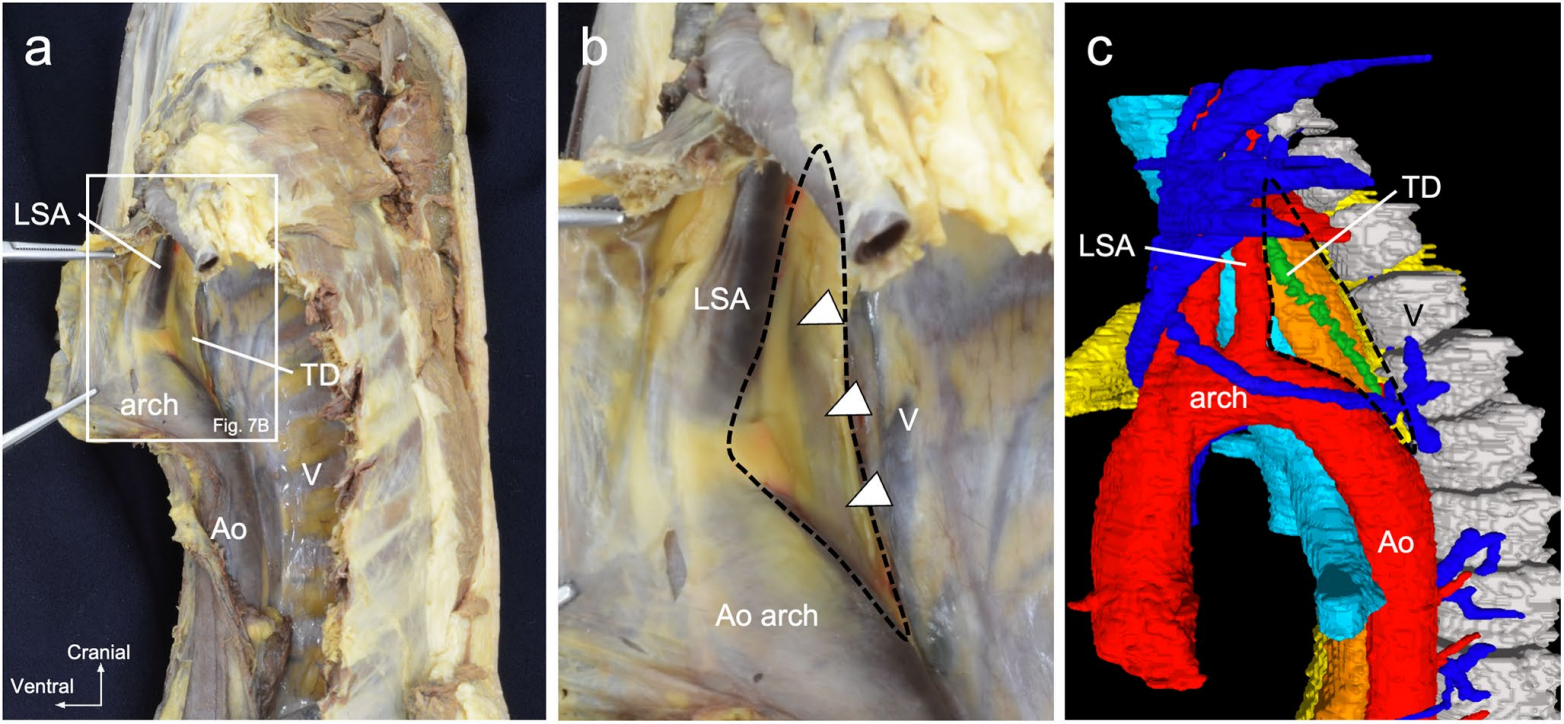
Poirier’s triangle in macroscopic anatomy and three-dimensional reconstruction image. Poirier’s triangle is the region in which the thoracic duct does not accompany the arteries. **a** Image of the mediastinum from the left side on macroscopic examination. **b** Magnified image of the box region in Fig. 7a. Poirier’s triangle, bordered by the dorsal edge of the left subclavian artery, cranial edge of the aortic arch, and ventral edge of the vertebral body, is observed. The thoracic duct ascends within Poirier’s triangle. **c** Poirier’s triangle in the three-dimensional image*Ao* aorta, *LSA* left subclavian artery, *TD* thoracic duct, *V* vertebra

Macroscopic anatomy, observation of anatomical sections, and three-dimensional reconstruction revealed an area superior to the aortic arch where the thoracic duct did not accompany the artery (Fig. 7a-c). After diverging from the aorta, the thoracic duct ascended into a triangular region bounded by the anterior vertebral margin, aortic arch, and left subclavian artery, accompanied by the left subclavian artery. In the triangular region, the thoracic duct, which was in contact only with the left pleura and surrounded by adipose tissues, was observed from the thoracic cavity through the translucent left pleura.

### Measurement of respiratory variations in the distance between arterial landmarks on CT images

**Fig. 8:**
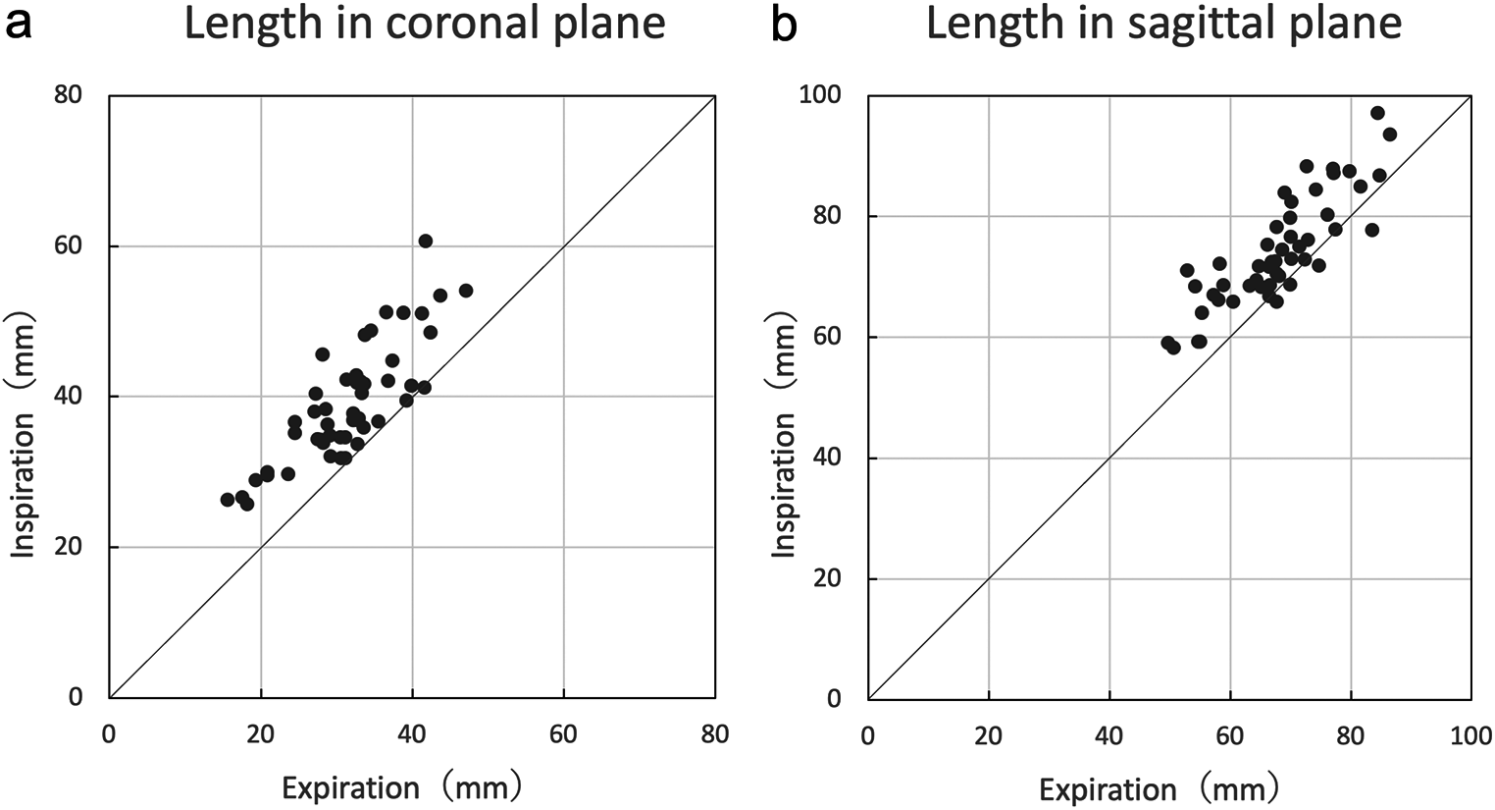
Variation of the thoracic duct route length during respiration. **a** Length in the coronal plane. The length is significantly greater during inspiration. **b** Length in the sagittal plane. The length is significantly greater during inspiration

The distance between the arterial landmarks in the coronal plane (Fig. 1a, A) was 39.03 ± 7.81 mm and 31.49 ± 7.01 mm during inspiration and expiration, respectively (Table 1). The distance in the sagittal plane (Fig. 1b, B) was 67.94 ± 9.08 mm and 74.16 ± 8.95 mm during inspiration and expiration, respectively. The difference between the inspiratory and expiratory distances (distance during inspiration -distance during expiration) was 7.53 ± 4.46 mm and 6.22 ± 5.00 mm in the coronal and the sagittal planes, respectively. The distance during inspiration was significantly longer than that during expiration (*p* < 0.001, Fig. 8).

**Table 1:** Length of the thoracic duct route on computed tomography images

	a. Coronal				b. Sagittal		
	Inspiration	Expiration	Difference		Inspiration	Expiration	Difference
Mean	39.03	31.49	7.53		74.16	67.94	6.22
Standard deviation	7.81	7.01	4.46		8.95	9.08	5.00

Difference=Inspiration-Expiration

## Discussion

In our examination, the thoracic duct accompanied the arteries in a large area of the mediastinum. It was accompanied by the aorta on the caudal side of the aortic arch. On the cranial side of the aortic arch, it moved anteriorly upward between the esophagus and left pleura, accompanied by the left common carotid and subclavian arteries, and joined the venous angle. The distance between the arterial landmarks in the upper mediastinum differed with respiration and was longer during inspiration.

The thoracic duct is the largest lymphatic duct in the human body. It originates from the cisterna chyli in the abdomen, ascends to the posterior mediastinum, and opens into the venous angle of the neck [12, 19]. Adachi classified variations of the thoracic duct into nine types based on their location and outflow position; type VI, which ascends on the right side of the aorta and opens on the left side, was the most common type [1]. In subsequent studies, although bifurcations, anastomoses, and plexiform variations were observed [6, 7, 12, 15], the type VI thoracic duct was the most common [2, 9, 13, 14]. In the present study, as in previous studies, the thoracic duct ascended on the right side of the aorta in the inferior mediastinum and along the left common carotid and subclavian arteries in the superior mediastinum and neck, and was thus close to the arteries for most of its course [2, 9, 13, 14]. These findings suggest that the basic route of the thoracic duct is along the arteries. A previous study reported that the thoracic duct showed a similar configuration when the descending aorta showed leftward displacement, suggesting that the course of the thoracic duct was strongly influenced by that of the descending aorta [10, 14].

Although many studies have reported a positional relationship between the thoracic duct and arteries, little is known about their relationship with the pleura. Tokairin et al. reported a membranous structure connecting the bilateral pleurae entering the mediastinum and separating the thoracic duct from the esophagus, suggesting that the thoracic duct also has a positional relationship with the pleurae [17]. This study revealed that the thoracic duct, arteries, and pleurae are located over a large area of the mediastinum. This anatomical finding provides insights into the mechanisms of lymphatic flow generation in the thoracic duct.

Lymph, like veins, is known to be compressed by the accompanying arteries and pushed out through valved vessels. Venous blood in the lower limbs flows into the valved venous vessels as it is pushed proximally by the pulsation of the accompanying arteries and contraction of the adjacent muscles [8, 16]. In all three methods–macroscopic examination, observation of anatomical sections, and three-dimensional reconstruction–the thoracic duct accompanied the arteries over a large area of the mediastinum, and the pleurae entered the mediastinum to sandwich the thoracic duct with the arteries. These results suggest that the pleurae, extending into the mediastinum, push the thoracic duct toward the arteries and create close contact between the thoracic duct and arteries, which promotes the generation of lymphatic flow by arterial pulsation. However, the thoracic duct is not accompanied by arteries on the cranial side of the aortic arch. Observing the mediastinum from the left thoracic cavity, a triangular space framed by the superior border of the aortic arch, posterior border of the left subclavian artery, and anterior border of the vertebral column was identified. This space has been reported as Poirier’s triangle [3, 18, 20]. Poirier’s triangle is an index of the thoracic duct ligation procedure via the thoracic cavity, and the thoracic duct ascending between the left pleura and the esophagus is often identified within the triangle. The Poirier’s triangle and thoracic duct within the triangle were observed in the present study by anatomical examination and three-dimensional reconstruction (Fig. 7a, b, black dotted line). The thoracic duct within the triangle was independent of the arteries.

Within Poirier’s triangle, it is difficult for the thoracic duct to receive the force for lymphatic flow from the arteries. The thoracic duct was attached to the descending aorta and left subclavian artery on the caudal and cranial sides of Poirier’s triangle, respectively. In contrast, the thoracic duct within Poirier’s triangle was surrounded by adipose tissue between the left pleura and esophagus with a loose connection. These results suggest that the thoracic duct within the Poirier’s triangle is mobile, which allows it to receive a force different from the arterial pulsation for lymphatic flow.

The thorax and diaphragm move significantly during respiration. During inspiration, the first rib to which the subclavian artery is attached is elevated, and the diaphragm to which the heart connected to the aorta is attached is lowered. Therefore, we hypothesized that the length of the thoracic duct changes with respiratory movement. Since observing the thoracic duct on plain CT images is challenging, we measured the distance between the arterial landmarks to estimate the thoracic duct length. The distances between the arterial landmarks were longer during inspiration than during expiration in both the coronal and sagittal planes. The changes in the distance between the arterial landmarks suggest that the length of the thoracic duct varies with respiration. Respiratory movement may have changed the relative position of the left subclavian artery and aorta, to which the thoracic duct is attached. Thus, the thoracic duct, superior to the aortic arch, may lengthen during inspiration and shorten during expiration.

Ultrasonographic observations by Hinton et al. showed that the thoracic duct diameter at the lymphovenous junction varied with respiration and was larger during inspiration. The thoracic duct terminal diameter was 1.830 ± 0.5957 mm and 1.640 ± 0.6509 mm during inspiration and expiration, respectively [5]. While Hinton et al. focused on the thoracic duct terminal diameter and respiration, the present study focused on the length of the thoracic duct route in the superior mediastinum and respiration. The results of these two studies suggest that during inspiration, both the thoracic duct length in the superior mediastinum and the thoracic duct terminal diameter increase, indicating that the thoracic duct in the superior mediastinum is stretched and thinned, and that the terminal diameter is enlarged as the lymph is squeezed out. These results suggest that the thoracic duct around the Poirier’s triangle is stretched and morphologically changed by respiratory movement to support the lymphatic flow.

The study shows the location of the thoracic duct, its position in relation to other structures, indicators on CT images, and respiratory variations in the morphology of structures in the mediastinum. These findings may lead to the prevention or early detection of thoracic duct injuries associated with mediastinoscopic and thoracoscopic procedures performed in esophageal surgery.

### Limitations

This study has several limitations. First, in the macroscopic examination, observation of anatomical sections, and three-dimensional reconstruction, we focused only on the thoracic duct that was visible macroscopically or in the viewer; thus, we did not consider the thoracic duct that was extremely thin or passed through unexpected areas. Additionally, the small sample size limited the ability to account for minor anatomical variations. Second, when measuring the thoracic duct on CT images, we used plain CT images to obtain both inspiratory and expiratory data, and it was difficult to identify the thoracic duct completely; therefore, we measured the length between the arteries to which the thoracic duct was attached, identified during macroscopic analysis. The thoracic duct length, lymphatic flow, and changes in intrapleural pressure were not measured. In future studies, the use of contrast imaging of the thoracic duct and measurements of lymphatic flow may provide further insights into the expansion and contraction of the thoracic duct and the generation of lymphatic flow.

## Conclusions

The thoracic duct is located close to the pleura and is accompanied by arteries over a wide area within the mediastinum. However, in the superior mediastinum, there is an area where the thoracic duct does not accompany the arteries and the length of the thoracic duct route is predicted to changes with respiration. These results suggest that the lymph flow of the thoracic duct is not only supported by the arterial pulsation but also by the expansion and contraction of the thoracic duct with respiratory movement.

## Data Availability

No datasets were generated or analysed during the current study.

## References

[1] Adachi B (1953) Der Ductus Thoracicus Der Japaner. Kenkyusha, Kyoto

[2] Akcali O, Kiray A, Ergur I, Tetik S, Alici E (2006) Thoracic duct variations may complicate the anterior spine procedures. Eur Spine J 15:1347–1351. 10.1007/s00586-006-0082-3 16544156 10.1007/s00586-006-0082-3 PMC2438572

[3] Caronia FP, Fatica F, Librizzi D, Fiorelli A (2019) Uniportal thoracoscopic thoracic duct clipping in Poirier’s triangle for postoperative chylothorax. Ann Thorac Surg 107:e415–e416. 10.1016/j.athoracsur.2018.09.062 30444992 10.1016/j.athoracsur.2018.09.062

[4] Chung BS, Park HS, Park JS, Hwang SB, Chung MS (2021) Sectioned and segmented images of the male whole body, female whole body, male head, and female pelvis from the visible Korean. Anat Sci Int 96:168–173. 10.1007/s12565-020-00562-y 32803722 10.1007/s12565-020-00562-y

[5] Hinton LR, O’Hagan LA, Griffiths AP, Clark AR, Phillips ARJ, Windsor JA, Mirjalili SA (2022) The effect of respiration and body position on terminal thoracic duct diameter and the lymphovenous junction: an exploratory ultrasound study. Clin Anat 35:447–453. 10.1002/ca.23801 34658062 10.1002/ca.23801

[6] Johnson OW, Chick JFB, Chauhan NR et al (2016) The thoracic duct: clinical importance, anatomic variation, imaging, and embolization. Eur Radiol 26:2482–2493. 10.1007/s00330-015-4112-6 26628065 10.1007/s00330-015-4112-6

[7] Kausel HW, Reeve TS, Stein AA, Stranahan A (1957) Anatomic and pathologic studies of the thoracic duct. J Thorac Surg 34:631–642 13476471

[8] Moore KL, Dalley AF, Agur AMR (2005) Clinically oriented anatomy, 5th edn. Lippincott Williams & Wilkins, Philadelphia

[9] Okuda I, Udagawa H, Takahashi J, Yamase H, Kohno T, Nakajima Y (2009) Magnetic resonance-thoracic ductography: imaging aid for thoracic surgery and thoracic duct depiction based on embryological considerations. Gen Thorac Cardiovasc Surg 57:640–646. 10.1007/s11748-009-0483-4 20013098 10.1007/s11748-009-0483-4

[10] Okuda I, Udagawa H, Hirata K, Nakajima Y (2011) Depiction of the thoracic duct by magnetic resonance imaging: comparison between magnetic resonance imaging and the anatomical literature. Jpn J Radiol 29:39–45. 10.1007/s11604-010-0515-0 21264660 10.1007/s11604-010-0515-0

[11] Park JS, Chung MS, Hwang SB, Lee YS, Har DH, Park HS (2005) Visible Korean human: improved serially sectioned images of the entire body. IEEE Trans Med Imaging 24:352–360. 10.1109/TMI.2004.842454 15754985 10.1109/tmi.2004.842454

[12] Plutecki D, Bonczar M, Wilk J et al (2024) The anatomy of the thoracic duct and cisterna chyli: a meta-analysis with surgical implications. J Clin Med 13:4285. 10.3390/jcm13154285 39124550 10.3390/jcm13154285 PMC11313251

[13] Rabattu PY, Sole Cruz E, El Housseini N et al (2021) Anatomical study of the thoracic duct and its clinical implications in thoracic and pediatric surgery, a 70 cases cadaveric study. Surg Radiol Anat 43:1481–1489. 10.1007/s00276-021-02764-z 34050781 10.1007/s00276-021-02764-z

[14] Rosenberger A, Abrams HL (1971) Radiology of the thoracic duct. Am J Roentgenol Radium Ther Nucl Med 111:807–820. 10.2214/ajr.111.4.807 4931468 10.2214/ajr.111.4.807

[15] Shimada K, Sato I (1997) Morphological and histological analysis of the thoracic duct at the jugulo-subclavian junction in Japanese cadavers. Clin Anat 10:163–172. 10.1002/(SICI)1098-2353(1997)10:3%3C163::AID-CA2%3E3.0.CO;2-V 9135884 10.1002/(SICI)1098-2353(1997)10:3<163::AID-CA2>3.0.CO;2-V

[16] Standring S (2016) Gray’s anatomy: the anatomical basis of clinical practice, 41st edn. Elsevier, New York

[17] Tokairin Y, Nagai K, Kawamura Y et al (2021) Histological study of the thin membranous dense connective tissue around the middle and lower thoracic esophagus, caudal to the bifurcation of the trachea. Gen Thorac Cardiovasc Surg 69:983–992. 10.1007/s11748-021-01615-3 33713025 10.1007/s11748-021-01615-3

[18] Tubbs RS, Noordeh N, Parmar A et al (2010) Reliability of Poirier’s triangle in localizing the thoracic duct in the thorax. Surg Radiol Anat 32:757–760. 10.1007/s00276-010-0681-x 20480366 10.1007/s00276-010-0681-x

[19] Williams P (1995) Gray’s anatomy: the anatomical basis of medicine and surgery, 38th edn. Churchill Livingstone, New York

[20] Worthington MG, De Groot M, Gunning AJ, Von Oppell UO (1995) Isolated thoracic duct injury after penetrating chest trauma. Ann Thorac Surg 60:272–274. 10.1016/0003-4975(95)00415-h 7646086 10.1016/0003-4975(95)00415-h

[21] You Y, Kim CY, Kim SK, Chung BS, Har D, Choi J, Park JS (2022) Advanced-sectioned images obtained by microsectioning of the entire male body. Clin Anat 35:79–86. 10.1002/ca.23795 34591338 10.1002/ca.23795

